# Organic Acids as Alternatives for Antibiotic Growth Promoters Alter the Intestinal Structure and Microbiota and Improve the Growth Performance in Broilers

**DOI:** 10.3389/fmicb.2020.618144

**Published:** 2021-01-14

**Authors:** Dong Dai, Kai Qiu, Hai-jun Zhang, Shu-geng Wu, Yan-ming Han, Yuan-yuan Wu, Guang-hai Qi, Jing Wang

**Affiliations:** ^1^Laboratory of Quality & Safety Risk Assessment for Animal Products on Feed Hazards (Beijing) of the Ministry of Agriculture & Rural Affairs, Feed Research Institute, Chinese Academy of Agricultural Sciences, Beijing, China; ^2^Trouw Nutrition Research & Development Centers, Amersfoort, Netherlands

**Keywords:** antibiotic, broiler, microbiota, organic acids, short-chain fatty acids

## Abstract

The present study aimed to investigate the effects of organic acids (OA) as alternatives for antibiotic growth promoters (AGP) on growth performance, intestinal structure, as well as intestinal microbial composition and short-chain fatty acids (SCFAs) profiles in broilers. A total of 336 newly hatched male Arbor Acres broiler chicks were randomly allocated into 3 dietary treatments including the basal diet [negative control (NC)], the basal diet supplemented with 5 mg/kg flavomycin, and the basal diet supplemented with OA feed additives. Each treatment had eight replicates with 14 birds each. The results showed that AGP and OA promoted growth during day 22–42 compared with the NC group (*P* < 0.05). OA significantly increased the jejunal goblet cell density and ileal villus height on day 42 compared with the NC group (*P* < 0.05). Meanwhile, OA up-regulated the mRNA expression of jejunal barrier genes (Claudin-3 and ZO-1) relative to the NC group (*P* < 0.05). Significant changes of microbiota induced by the OA were also found on day 42 (*P* < 0.05). Several SCFAs-producing bacteria like Ruminococcaceae, Christensenellaceae, and Peptococcaceae affiliated to the order Clostridiales were identified as biomarkers of the OA group. Higher concentrations of SCFAs including formic acid and butyric acid were observed in the cecum of OA group (*P* < 0.05). Simultaneously, the abundance of family Ruminococcaceae showed highly positive correlations with the body weight and mRNA level of ZO-1 on day 42 (*P* < 0.05). However, AGP supplementation had the higher mRNA expression of Claudin-2, lower goblet cell density of jejunum, and decreased Firmicutes to Bacteroidetes ratio, suggesting that AGP might have a negative impact on intestinal immune and microbiota homeostasis. In conclusion, the OA improved growth performance, intestinal morphology and barrier function in broilers, which might be attributed to the changes of intestinal microbiota, particularly the enrichment of SCFAs-producing bacteria, providing a more homeostatic and healthy intestinal microecology.

## Introduction

Subtherapeutic levels of antibiotics have been extensively used in the commercial poultry production to maintain health and to improve growth performance and feed conversion efficiency (Dibner and Richards, 2005; Markowiak and Śliżewska, 2018). However, the use of antibiotics as feed additives has been restricted or even forbidden in many countries because of the increasing concerns on the persistence of antibiotic residues and the transmission of antibiotic resistance, both of which pose a potential threat to human health (Gadde et al., 2017; Muaz et al., 2018). Antibiotics removal has led to a series of problems such as decreased growth performance and increased incidence of intestinal diseases (Dibner and Richards, 2005). Optimal intestinal health is important for gut barrier function, microflora, and digestion and absorption of nutrients, contributing to improved growth performance. Therefore, it is necessary to develop novel feed additives substituting antibiotics to maintain intestinal health in the poultry production (Salaheen et al., 2017).

Organic acids (OA), which are considered to be organic carboxylic acid of the general structure R-COOH, have long been utilized to preserve feed and have been used as feed additives in the broilers production for more than three decades (Polycarpo et al., 2017). Beneficial effects of OA on growth performance of poultry have already been described in numerous studies (Paul et al., 2007; Ghazalah et al., 2011; Khan and Iqbal, 2016). Although it is clear that OA could reduce the pH in the gastrointestinal tract, increase the activity of digestive enzymes, and improve the apparent ileal digestibility of chickens (Hernández et al., 2006; Garcia et al., 2007; Ao et al., 2009), and have been considered as one of the most promising alternatives for antibiotic growth promoters (AGP) (Suiryanrayna and Ramana, 2015), the specific mechanism by which OA improve the growth performance has yet to be illuminated.

Intestinal microbiota plays an important role in digestion, barrier and immune function of chickens, contributing to subsequent improvement in the growth performance (Pan and Yu, 2014; Pandit et al., 2018). It is widely accepted that AGP boost animal growth mainly through reducing pathogenic bacteria and modulating gastrointestinal microbiota (Diarra and Malouin, 2014; Gadde et al., 2017). Similarly, antibacterial properties of OA against poultry pathogens have also been found using conventional molecular ecology techniques such as the cultures and denaturing gradient gel electrophoresis fingerprints in previous studies (Nava et al., 2009; Liu et al., 2017; Palamidi and Mountzouris, 2018). Nevertheless, they showed some inconsistent effects on the abundance of some designated intestinal bacteria (e.g., *Lactobacillus*) (Liu et al., 2017; Li et al., 2018; Palamidi and Mountzouris, 2018; Hu et al., 2019), which might be caused by the differences in the sensitivities and specificities of detection techniques besides the composition and concentrations of OA used. The advent of high-throughput sequencing of 16S rRNA gene amplicons enabled us to study the composition and diversity of microbiota including rare species more sensitively. Li et al. (2018) have elucidated the effects of OA on the microbial composition with the sequencing-based technique in weaned pigs. Nevertheless, studies about the effects of OA on the modulation of intestinal microbiota in broiler chickens remain limited and it requires further investigation in order to better explain the potential influence of dietary OA supplementation on the growth performance and intestinal health.

Therefore, the objective of the current study was initially to determine the effects of OA in the feed on the growth performance, intestinal morphology, short chain fatty acids (SCFAs), and expression of barrier-related genes in the intestine. Subsequently, the changes in cecal microbiota compositions were characterized by high throughput sequencing, and the correlations between the dominant bacteria with growth performance and intestinal barrier were analyzed. Our findings may contribute to exploring the regulatory role of OA on the intestinal health and growth performance of poultry, and expand our knowledge concerning application of OA and assessment of the AGP alternatives in animal feed.

## Materials and Methods

### Animal Experiments

A total of 336 newly hatched male Arbor Acres broiler chicks (Beijing Huadu Broiler Company, Beijing, China) were randomly allocated into 3 dietary treatments including the basal diet [negative control (NC)], active flavomycin components 5 mg/kg basal diet [positive control (PC), Phibro Animal Health Co., Ltd.], and the basal diet supplied with OA feed additives (Selko B.V., Netherlands). Each treatment had 8 replicates with 14 birds each. The diet was formulated to meet or exceed National Research Council (National Research Council, 1994) guidelines (Table 1). The OA feed additive is a synergistic blend of free and buffered OA mainly based on formic acid (≥11%), acetic acid (≥5.1%), propionic acid (≥10%), and ammonium formate (≥13%) on silica carrier and added to the basal diet at 0.3% (day 0–21) and 0.2% (day 22–42). Both diet and water were supplied *ad libitum* in pellet form and by nipple drinkers, respectively. All management of birds in this study was according to the guideline of raising AA broilers (Delezie et al., 2012). The experiment lasted for 42 days and samples were collected at the end of the experiment.

**TABLE 1:** Composition and nutrient levels of basal diet (air-dry basis).

**Item**	**Starting phase (day 0–21)**	**Growing phase (day 22–42)**
**Ingredients,%**		
Corn	57.90	60.70
Soybean meal	34.85	30.85
Soybean oil	2.53	4.72
Dicalcium phosphate	2.27	1.82
Limestone	0.82	0.70
Salt	0.35	0.35
*DL*-Methionine	0.34	0.20
*L*-lysine-HCl	0.31	0.04
Vitamin Premix^a^	0.02	0.02
Mineral Premix^b^	0.20	0.20
Choline chloride (50%)	0.10	0.10
Corn starch	0.30	0.20
Phytase (5,000 U/g)	0.01	0.01
Total	100.00	100.00
**Nutrient levels**		
AME (MJ/kg)	12.35	13.18
Crude protein,%	22.00	20.00
Calcium,%	1.00	0.90
Available phosphorus,%	0.50	0.40
Lysine,%	1.27	1.05
Methionine,%	0.55	0.48
Methionine + cystine,%	0.94	0.84

*^*a*^The vitamin premix supplied the following per kg of complete feed: vitamin A, 12 500 IU; vitamin D3, 2500 IU; vitamin K3, 2.65 mg; vitamin B1, 2 mg; vitamin B2, 6 mg; vitamin B6, 2.5 mg; vitamin B12, 0.025 mg; vitamin E, 30 IU; choline, 750 mg; biotin, 0.0325 mg; folic acid, 1.25 mg; pantothenic acid, 12 mg; niacin, 50 mg.^*b*^The mineral premix supplied the following per kg of complete feed: Cu, 8 mg; Zn, 75 mg; Fe, 80 mg; Mn, 100 mg; I, 0.35 mg, Se, 0.15 mg.*

### Growth Performance Measurement

The growth performance, including body weight (BW), average daily gain (ADG), average daily feed intake (ADFI), and feed conversion ratio (feed: gain, F/G), was recorded at the phases of day 0–21, day 22–42, and day 0–42.

### Intestinal Morphology and Goblet Cell Density

Segments (about 2 cm) of the middle of duodenum, jejunum, and ileum were collected and fixed in 10% neutral-buffered formalin for morphology on day 42. After wash, dehydration, clarification, and paraffin embedding procedures, a total of 5 μm thickness cut with serial sections was placed on glass slides to be deparaffinized in xylene, rehydrated, stained with hematoxylin and eosin and fixed with neutral balsam. The villus height (VH) and crypt depth (CD) were determined using an image processing and analyzing system (Inverted microscope: NIKON CI-S, Tokyo, Japan; Imaging system: NIKON DS-U3, Tokyo, Japan).

Goblet cells were the major source of mucin and were studied by intestinal cross-sections stained with Alcian Blue and periodic acid-Schiff reagent (Sigma Chemical Co., St Louis, MO, United States). The goblet cells were analyzed in cross-sections of the villi. Pictures were taken and analyzed, respectively, using an Olympus DP12 CCD digital camera and Image-Pro Plus 6.0 software (Media Cybernetics, Bethesda, MD, United States). The goblet cell density (GC) was defined as the goblet cell count per 100 μm villus length. The average value of the goblet cell density of three villi from each sample was used.

### Gene Expression of Tight Junction Proteins in Jejunum

Total RNA was extracted from jejunal tissues with the TRIzol reagent (Tiangen Biotech Co., Ltd., Beijing, China) according to manufacturer’s instructions. The concentration and integrity of RNA was analyzed using a NanoDrop 2000 spectrophotometer (Thermo Fisher Scientific, Waltham, MA, United States) and agarose-ethidium bromide electrophoresis, respectively. Quantification included reverse transcription and PCR with the FastQuant RT kit (KR106, Tiangen Biotech Co., Ltd., Beijing, China) and each RT reaction contained of 1 μg RNA. PCR was performed using the Light Cycler 480 Real-time PCR Instrument (Roche Diagnostics Ltd., Basel, Switzerland) and the SuperReal PreMix Plus kit (SYBR Green, KR106, Tiangen Co., Beijing, China). PCR reactions were performed using the C1000 thermal cycler (CFX-96 real-time PCR detection systems, Bio-Rad Laboratories, Inc., CA, United States) and each sample was measured in duplicate. The Table 2 showed primers sequences used in this study. The relative mRNA expression levels were normalized to avian β-actin by the 2^–ΔΔCt^ method (Livak and Schmittgen, 2001).

**TABLE 2:** Primer sequences of target and reference genes involved in the intestinal barrier function.

**Gene name**	**Forward primer (5′ to 3′)**	**Reverse primer (5′ to 3′)**	**GenBank number**
Claudin-1	CATACTCCTGGGTCTGGTTGGT	GACAGCCATCCGCATCTTCT	NM_001013611.2
Claudin-2	CTGCTCACCCTCATTGGA	AACTCACTCTTGGGCTTCTG	NM_001277622.1
Claudin-3	GCTCTGCCGTTACCAGCTACG	CTGCACACAGCTCTCCTGGCAAC	NM_204202.1
Claudin-4	GAAGCGCTGAACCGATACCA	TGCTTCTGTGCCTCAGTTTCC	AY435420.1
Occludin	ACGGCAGCACCTACCTCAA	GGGCGAAGAAGCAGATGAG	NM_205128.1
Zonula occludens-1	TGTAGCCACAGCAAGAGGTG	CTGGAATGGCTCCTTGTGGT	XM_413773.4
β-actin	GAGAAATTGTGCGTGACATCA	CCTGAACCTCTCATTGCCA	EF667345.1

### SCFAs Profiling

A total of 100 mg of frozen cecal samples were used to determine SCFAs (formic acid, acetic acid, propionic acid, and butyric acid). Briefly, cecal samples were homogenized with 1 mL of sterile PBS followed by centrifugation (12000 rpm, 10 min at 4°C). Then, a 500 μL of the supernatant was diluted with 100 μL of 25% (w/v) metaphosphoric acid solution containing crotonic acid. The mixture was incubated at −20°C for 24 h and then centrifuged (12000 rpm, 10 min at 4°C). The extracted solution was filtered with a 0.22 μm syringe filter and analyzed SCFAs using a gas chromatograph (Shimadzu GC-2010 ATF instrument) equipped with a capillary column (30 m × 0.25 mm × 0.5 μm). The N_2_ was used for carrier gas (12.5 Mpa, 18 mL/min). The temperature of the injector and detector was 180°C, and the column was gradually heated from 80 to 170°C at a rate of 5°C min^–1^.

### DNA Extraction and PCR Amplification of 16S rRNA Gene Sequences

Microbial DNA was extracted from 300 mg cecal content samples taken from all groups using the E.Z.N.A Soil DNA Kit (Omega Bio-tek, Norcross, GA, United States) according to manufacturer’s instructions. The V3–V4 regions of bacterial 16S rDNA sequences were amplified using primer 338F (5′-ACTCCTACGGGAGGCAGCA-3′) and 806R (5′-ACTCCTACGGGAGGCAGCA-3′) according to the method described previously (Dai et al., 2020). Purified amplicons were pooled in equal amounts and paired-end sequenced (2 × 250 bp) throughput analysis was performed at Shanghai Majorbio Bio-Pharm Technology Co., Ltd., using the Illumina MiSeq platform. The raw reads were deposited into the NCBI Sequence Read Archive (SRA) database (Accession Number: PRJNA665111).

### Statistical Analysis

Data analysis was performed using SPSS version 19.0 for Windows (SPSS Inc., Chicago, IL, United States). The replicate (each replicate included one cage) was the experimental unit to analyze performance, and individual bird as the experimental unit to analyze other parameters. The normality of data was initially tested using the Shapiro–Wilk test. Data were analyzed using one-way ANOVA and means were compared using Duncan’s multiple range test. Differences were considered statistically significant at *P* < 0.05, and a tendency toward significance considered at 0.05 ≤ *P* < 0.10. Data were expressed as mean and pooled SEM.

For microbiota profiling, raw pair-end sequences were demultiplexed and quality-filtered using The Quantitative Insights Into Microbial Ecology (QIIME, version 1.17) (Edgar, 2010). Sequences with length shorter than 150 bp, average Phred scores lower than 20 were filtered through (Dai et al., 2020). The effective reads were clustered into operational taxonomic units (OTUs) based on the 97% similarity. Classification of OTUs at various taxonomic levels were implemented using the Greengenes database. For rarefaction curves and α-diversity analysis were calculated using QIIME (Caporaso et al., 2010). β-diversity was estimated using principal coordinate analysis (PCoA) and partial least squares discriminant analysis (PLS-DA). The results were plotted using “vegan” and “ggplot2” package in R software (Version 3.4.4). The significance of microbial community differences among groups was assessed using ANOSIM with R package “vegan” (Warton et al., 2012). Before Linear discriminant analysis (LDA) combined effect size (LEfSe) estimate the impact of the abundance of bacteria on the difference effect of bacteria among groups (LDA > 3, *P* < 0.05), non-parametric factorial Kruskal–Wallis sum-rank test was employed to explore the differences in the relative abundances of bacteria among groups (Segata et al., 2011). Correlations were analyzed using spearman correlation with the pheatmap package (*P* < 0.05).

## Results

### Growth Performance

The effects of dietary supplementation with OA on growth performance of broilers were showed in Table 3. There were no significant differences in performance among groups during day 0–21 (*P* > 0.05). Higher ADG on day 22–42 (*P* = 0.039) and whole phase (*P* = 0.020) were observed in birds fed with the AGP and OA compared with the NC group. The two groups also had greater final BW than the NC group (*P* = 0.022).

**TABLE 3:** Effects of dietary supplementation with organic acids on growth performance of broilers.

**Items^1^**	**NC**	**PC**	**OA**	**SEM**	***P*-value**
**Starter phase (day 0–21)**					
BW day 21 (g)	974.52	990.29	963.50	8.54	0.456
ADG (g)	43.98	44.88	43.31	0.40	0.283
ADFI (g)	54.73	55.40	53.89	0.64	0.647
F/G	1.24	1.24	1.25	0.01	0.897
**Grower phase (day 22–42)**					
BW day 42 (kg)	2.54^b^	2.68^a^	2.63^a^	0.02	0.022
ADG (g)	74.53^b^	79.21^a^	78.01^a^	0.77	0.039
ADFI (g)	128.35	136.15	133.95	1.50	0.109
F/G	1.72	1.72	1.72	0.01	0.979
**Whole phase (day 0–42)**					
ADG (g)	57.93^b^	61.28^a^	59.98^a^	0.50	0.020
ADFI (g)	88.82	93.90	91.77	0.92	0.091
F/G	1.53	1.53	1.53	0.01	0.985

*^*a,b*^Means within a row with no common superscript differ significantly (*n* = 8; *P* < 0.05).^1^NC, negative control supplied with the basal diets; PC, positive control supplied with the basal diets and 5 mg/kg flavomycin; OA, the basal diets supplied with 0.3% (day 0–21) and 0.2% (day 22–42) organic acids feed additives; BW, body weight; ADG, average daily gain; ADFI, average daily feed intake; F/G, feed conversion ratio (feed: gain, g: g).*

### Intestinal Morphology and Goblet Cell Density

**FIGURE 1:**
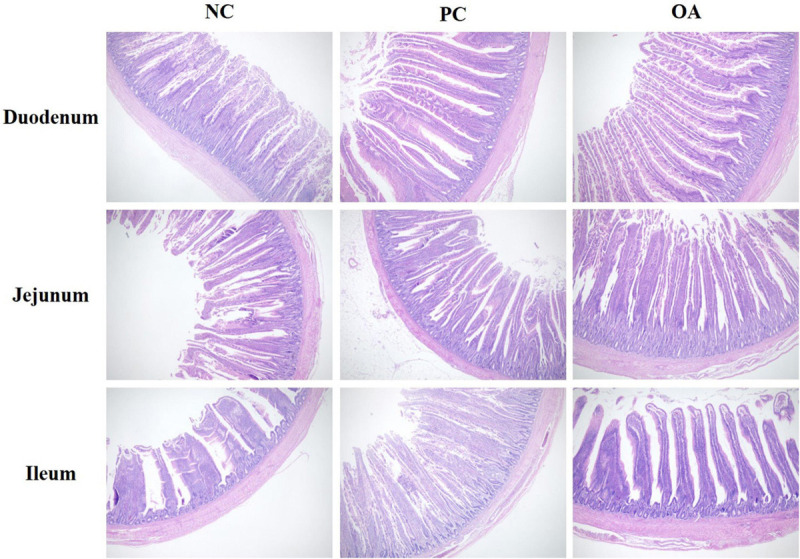
Intestinal (duodenum, jejunum, and ileum) morphological structure in broilers on day 42. The pictures were observed at 100× magnification (*n* = 8). NC, negative control supplied with the basal diets; PC, positive control supplied with the basal diets and 5 mg/kg flavomycin; OA, the basal diets supplied with 0.3% (day 0–21) and 0.2% (day 22–42) organic acids feed additives.

The light micrographs of small intestine morphology were exhibited in Figure 1. We could clearly find that OA and AGP increased the VH of the ileum. Moreover, the results were statistically confirmed by the data shown in Table 4. The VH of ileum was greater in PC and OA groups compared with the NC group (*P* = 0.028), and showed no difference between the PC and OA groups (*P* > 0.05). In addition, the OA group increased the goblet cell density of jejunum significantly compared with the PC group (*P* = 0.035). However, no significant differences in morphology and goblet cell count of duodenum were observed on day 42 (*P* > 0.05).

**TABLE 4:** Effects of dietary supplementation with organic acids on the intestinal morphology and goblet cell density of broilers on day 42.

**Items^1^**	**NC**	**PC**	**OA**	**SEM**	***P*-value**
**Duodenum**					
VH (μm)	1703.66	1646.10	1807.55	49.51	0.426
CD (μm)	225.83	237.50	201.30	7.11	0.103
VH/CD	7.64	7.21	9.06	0.36	0.089
GC	20.48	17.78	20.12	1.00	0.511
**Jejunum**					
VH (μm)	942.83	1156.07	1106.70	39.11	0.073
CD (μm)	219.39	248.25	209.27	8.38	0.133
VH/CD	4.70	4.59	5.58	0.24	0.204
GC	17.51^ab^	12.73^b^	21.48^a^	1.17	0.035
**Ileum**					
VH (μm)	584.13^b^	741.09^a^	766.86^a^	32.26	0.028
CD (μm)	127.86	142.88	126.24	4.84	0.302
VH/CD	5.01	5.10	5.24	0.17	0.874
GC	20.12	26.06	26.48	1.25	0.074

*^*a,b*^Means within a row with no common superscript differ significantly (*n* = 8; *P* < 0.05).^1^NC, negative control supplied with the basal diets; PC, positive control supplied with the basal diets and 5 mg/kg flavomycin; OA, the basal diets supplied with 0.3% (day 0–21) and 0.2% (day 22–42) organic acids feed additives; VH, villus height; CD, crypt depth; VH/CD, the ratio of VH to CD; GC, goblet cell density (n/100 μm villi).*

### Gene Expression of Tight Junction Proteins in Jejunum

**FIGURE 2:**
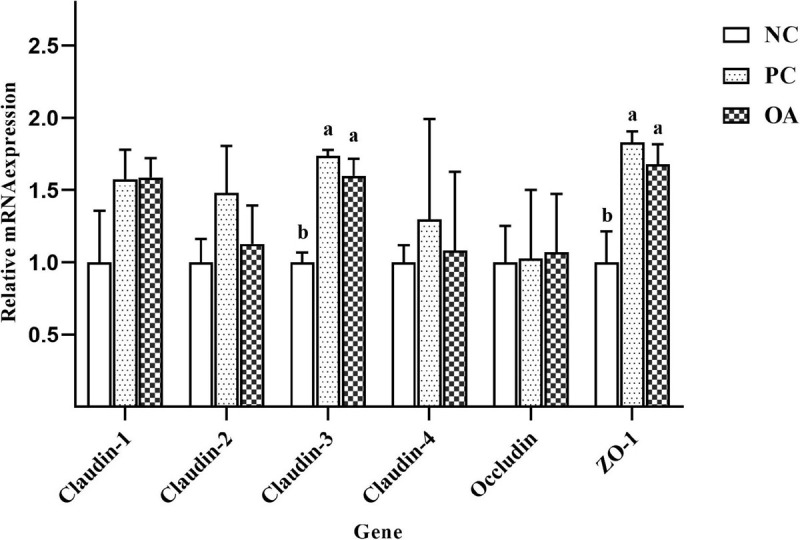
Effects of dietary supplementation with organic acids on the relative mRNA expressions of jejunal tight junction protein genes of broilers on day 42. ^*a,b*^Values at the same index with no common superscripts differ significantly (*n* = 8; *P* < 0.05). NC, negative control supplied with the basal diets; PC, positive control supplied with the basal diets and 5 mg/kg flavomycin; OA, the basal diets supplied with 0.3% (day 0–21) and 0.2% (day 22–42) organic acids feed additives; ZO-1, Zonula occludens-1.

To further investigate the effects of OA on the intestinal barrier integrity, relative mRNA expressions of jejunal tight junction proteins including Claudin-1, Claudin-2, Claudin-3, Claudin-4, Occludin, and Zonula occludens-1 (ZO-1) were measured (Figure 2). We found that OA and AGP could up-regulate relative mRNA expressions of Claudin-3 and ZO-1 significantly compared with the NC group (*P* < 0.05). Similarly, higher relative mRNA expression of Claudin-1 was also observed in PC and OA groups compared with the NC group (*P* = 0.076). In addition, there was an increasing tendency in Claudin-2 mRNA expression in the PC group compared with NC and OA groups (*P* = 0.083). However, there were no significant differences in relative mRNA expressions of Claudin-4 and Occludin among groups (*P* > 0.05).

### SCFAs Profiling

**FIGURE 3:**
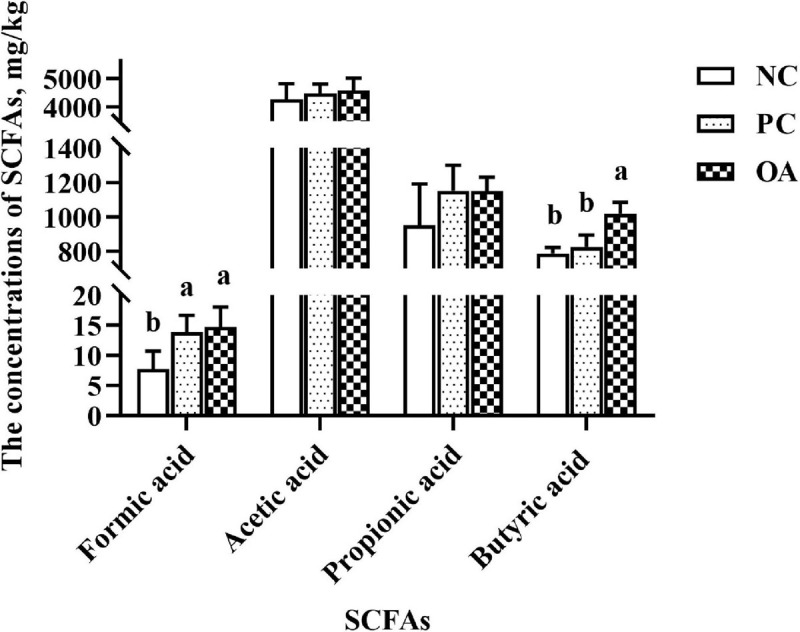
Effect of dietary supplementation with organic acids on the concentrations of cecal short-chain fatty acids of broilers on day 42. ^*a,b*^Values at the same index with no common superscripts differ significantly (*n* = 8; *P* < 0.05). NC, negative control supplied with the basal diets; PC, positive control supplied with the basal diets and 5 mg/kg flavomycin; OA, the basal diets supplied with 0.3% (day 0–21) and 0.2% (day 22–42) organic acids feed additives; SCFAs, short-chain fatty acids.

SCFAs, major carbohydrate fermentation products of gut microbiota, serving as indicators of microbial activity were detected and quantified here (Figure 3). The higher concentration of formic acid were observed in PC and OA groups (*P* < 0.05). Additionally, OA supplementation could also increase the concentration of butyric acid in the cecum compared with NC and PC groups (*P* < 0.05). However, no significant differences were observed in concentrations of acetic acid and propionic acid (*P* > 0.05).

### Cecal Microbiota Analysis by 16S rDNA

**FIGURE 4:**
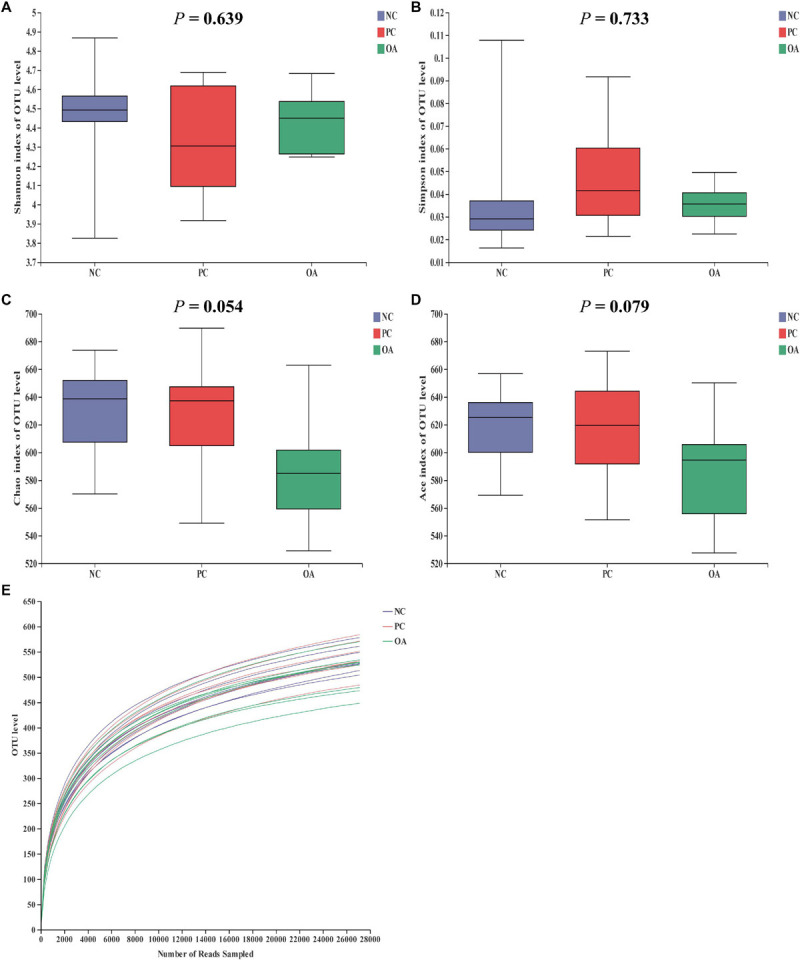
Effects of dietary supplementation with organic acids on the cecal microbial diversity and rarefaction curve of broilers on day 42. **(A–D)** were microbial diversity results and **(E)** was rarefaction curve. NC, negative control supplied with the basal diets; PC, positive control supplied with the basal diets and 5 mg/kg flavomycin; OA, the basal diets supplied with 0.3% (day 0–21) and 0.2% (day 22–42) organic acids feed additives.

**FIGURE 5:**
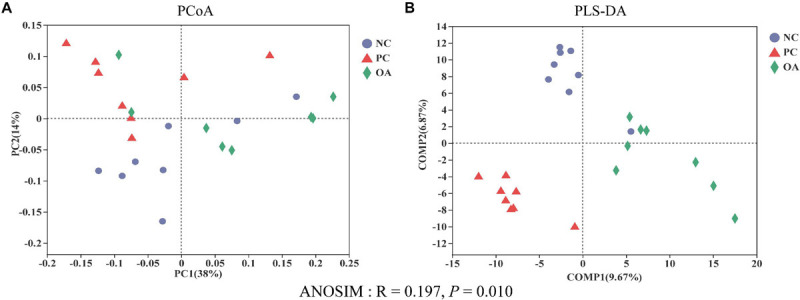
Effects of dietary supplementation with organic acids on the cecal microbial β-diversity of broilers on day 42. **(A)** principal coordinate analysis (PCoA) based on based on weighted UniFrac distance calculated from OTU abundance matrix; **(B)** partial least squares discriminant analysis (PLS-DA); Analysis of similarities (ANOSIM) of weighted UniFrac distances. NC, negative control supplied with the basal diets; PC, positive control supplied with the basal diets and 5 mg/kg flavomycin; OA, the basal diets supplied with 0.3% (day 0–21) and 0.2% (day 22–42) organic acids feed additives.

After filtering, an average of 59788 reads per sample was obtained. First, sequencing depths were examined by plotting the rarefaction curve for richness and the numbers of shared OTUs (Figure 4). Most of the samples reached plateaus, indicating that sampling depth was adequate. There was no difference in the α-diversity of the intestinal microbiota among groups (*P* > 0.05). β-diversity analysis were performed to compare the overall microbial profiles of all groups as displayed in Figure 5. PCoA analysis was firstly performed to present a holistic perception of the microbiota via weighted UniFrac distance metric. Results for PCoA visually showed that the groups were mainly scattered into three clusters. Moreover, PLS-DA plot defined groups where samples from different groups occupied distinct positions, which illustrated the microbiota compositions were quite dissimilar to each group. Results were also supported by statistics obtained from ANOSIM analysis (*R* = 0.197, *P* = 0.010).

**FIGURE 6:**
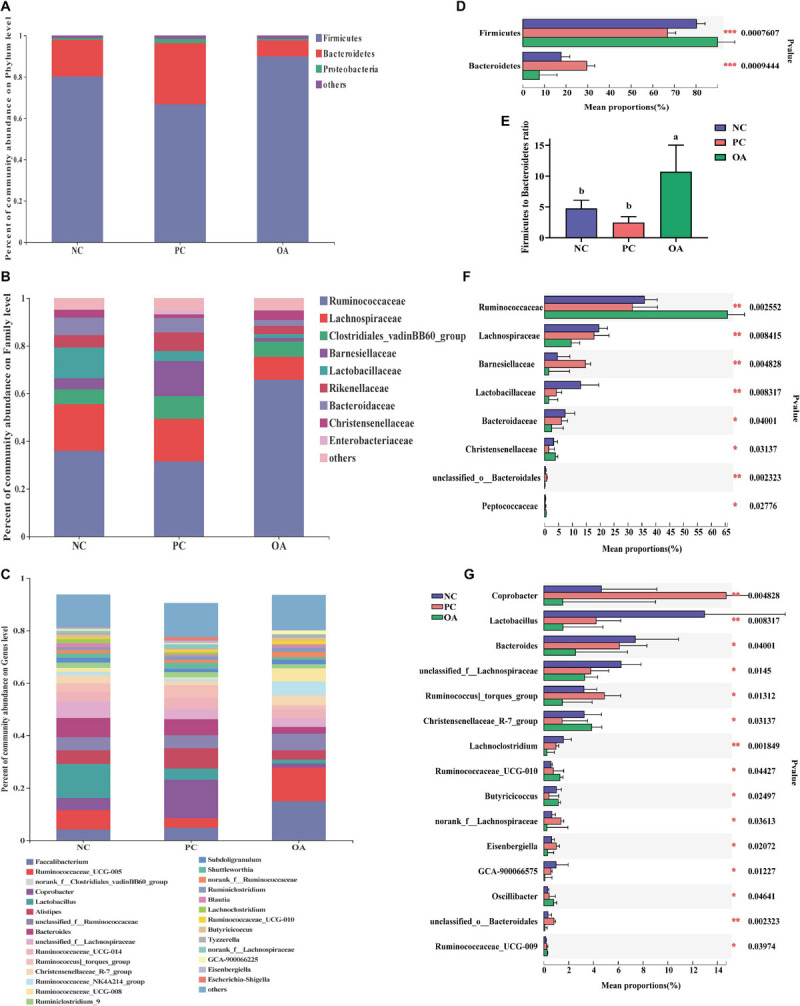
Compositions of cecal microbiota and differential species identified of broilers at phylum, family, and genus level on day 42. **(A–C)** were the bacterial community compositions at phylum, family, and genus level, respectively; **(D,F,G)** were significantly differential bacteria at phylum family and genus level, respectively and **(E)** was the ratio of the abundance of Firmicutes to Bacteroidetes. ^*a,b*^Values at the same index with no common superscripts differ significantly (*n* = 8; *P* < 0.05). NC, negative control supplied with the basal diets; PC, positive control supplied with the basal diets and 5 mg/kg flavomycin; OA, the basal diets supplied with 0.3% (day 0–21) and 0.2% (day 22–42) organic acids feed additives.

To assess the differences induced by OA in the cecal microbiota, taxonomic compositions were analyzed at phyla, family, and genus levels (Figure 6). Firmicutes and Bacteroidetes, the major phyla in cecal bacterial community, were differentially abundant among groups. Broilers fed with the OA diet were characterized by higher relative abundance of Firmicutes (89.95%: 80.19%, *P* = 0.001) and lower abundance of Bacteroidetes (7.69%: 17.73%, *P* = 0.001) compared with the NC group, thus leading to a higher Firmicutes to Bacteroidetes ratio (Figure 6E, *P* = 0.001). However, the opposite results were found in the PC group. The relative abundance of Ruminococcaceae and Peptococcaceae in the OA group were higher than that in the NC and PC groups (*P* = 0.003, *P* = 0.028, respectively), whereas the relative abundance of Lachnospiraceae, Barnesiellaceae, Lactobacillaceae, and Bacteroidaceae was lower than that in other groups (*P* = 0.008, *P* = 0.005, *P* = 0.008, *P* = 0.040, respectively). Compared with NC and PC groups at the genus level, *Faecalibacterium*, *Ruminococcaceae_UCG-005*, *Ruminococcaceae_NK4A214_group*, *Ruminococcaceae_UCG-010* were increased by 1.69 − 5.24 fold by the OA treatment (14.97%: 3.61% and 3.06%, 12.83%: 7.40% and 3.57%, 5.24%: 1.19% and 1.00%, 1.29%: 0.58% and 0.76%, respectively), whereas *Coprobacter*, *Lactobacillus*, *Bacteroides*, and *Lachnoclostridium* were decreased by 2.38 − 9.68 fold (1.52%: 4.64% and 14.71%, 1.55%: 12.96% and 4.22%, 2.56%: 7.38% and 6.10%, 0.24%: 0.98% and 1.58%, respectively).

**FIGURE 7:**
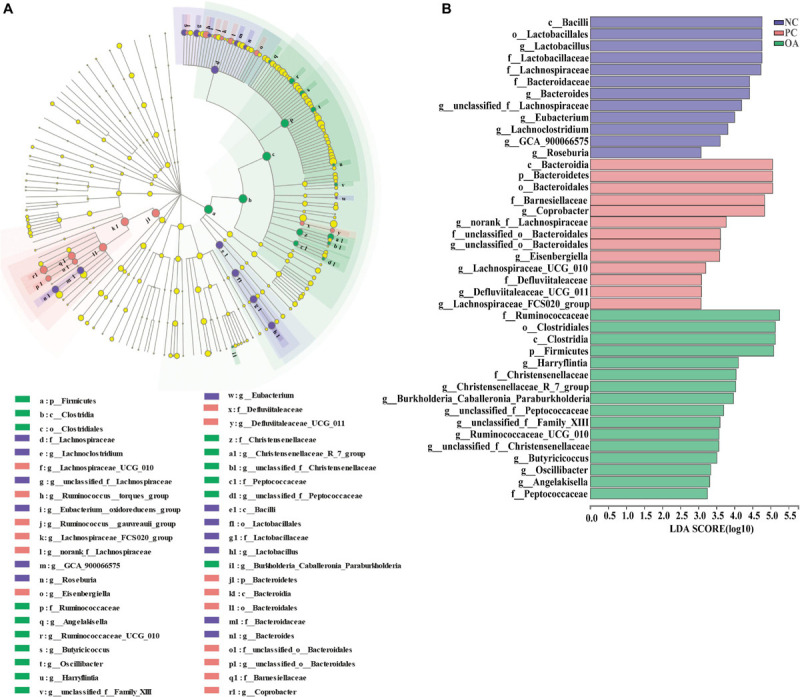
Linear discriminant analysis effect size (LEfSe) identified the most differentially abundant taxa enriched in cecal microbiota of broilers among the groups on day 42. **(A)** cladogram generated from LEfSe analysis; **(B)** histogram of the LDA scores computed for features differentially abundant (LDA > 3, *P* < 0.05). NC, negative control supplied with the basal diets; PC, positive control supplied with the basal diets and 5 mg/kg flavomycin; OA, the basal diets supplied with 0.3% (day 0–21) and 0.2% (day 22–42) organic acids feed additives.

In addition, the identifications of the key phylotypes responsible for differentiation among groups were also explored by the LEfSe method, and were represented by cladograms (Figure 7A) and scores bar-charts (Figure 7B). On day 42, the phyla of Firmicutes was enriched in the OA group whereas Bacteroidetes was found to be enriched in the PC group. At the family level, the cecal contents of the birds in the OA group were characterized by abundances of Ruminococcaceae (*Harryflintia*, *Ruminococcaceae_UCG_010*, *Butyricicoccus*, *Oscillibacter*, and *Angelakisella*), Peptococcaceae (*unclassified_f_Peptococcaceae*), and Christensenellaceae (*Christensenellaceae_R_7_group*) belonging to the order Clostridiales of Class Clostridia. Besides, we found that Lachnospiraceae (*Lachnospiraceae_UCG_010*, *Lachnospiraceae_FCS020_group*, *norank_f_Lachnospiraceae*, and *Eisenbergiella*), and Barnesiellaceae (*Coprobacter*) were linked to the increased relative abundance in the PC group. However, *Lactobacillus* was observed to be enriched only in the NC group.

### Correlations Between Microbiota and Growth Performance and Tight Junction Protein mRNA Expressions

**FIGURE 8:**
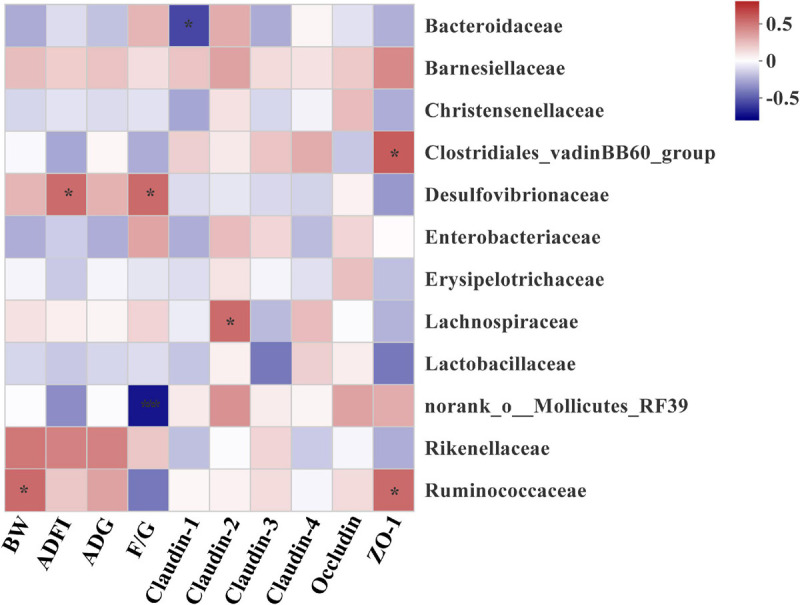
Heatmap of spearman’s correlation between intestinal microbiota at family level and growth performance and tight junction protein mRNA expressions of broilers on day 42. The colors range from blue (negative correlations) to red (positive correlations). Significant correlations are noted by 0.01 < *P* ≤ 0.05*, 0.001 < *P* ≤ 0.01**. BW, body weight; ADG, average daily gain; ADFI, average daily feed intake; F/G, feed conversion ratio (feed: gain, g: g); ZO-1, Zonula occludens-1.

To explore the bacteria associated with growth performance and intestinal barrier function, correlations between abundance of microbiota and the expressions of intestinal tight junction protein gene were analyzed based on the spearman’s correlation coefficients (Figure 8). The abundance of family Ruminococcaceae and Desulfovibrionaceae were showed highly positive correlations with the BW and ADFI, respectively (*P* < 0.05). However, the significant positive correlation between Desulfovibrionaceae and F/G was also determined from the heatmap (*P* < 0.05). In addition, the heatmap reflected that the abundance of Ruminococcaceae and Clostridiales_vadinBB60_group were positively correlated with the mRNA expression of ZO-1 (*P* < 0.05). And Lachnospiraceae family had a negative correlation with the mRNA expression of Claudin-2 (*P* < 0.05). In contrast, the significant positive correlation between Bacteroidaceae and the mRNA expression of Claudin-1 was also observed (*P* < 0.05).

## Discussion

Recently, with the legislation in regulations about restricting the use of AGP and the increasing concerns of consumers on AGP-free commercial poultry foods, there has been an intensified demand for AGP alternatives. Due to the high biological safety and beneficial functions, OA have been widely used in the field of animal feed (Polycarpo et al., 2017). Previous studies indicated that the addition of OA as substitutes for AGP to the broilers’ diet obviously improved the growth performance (Hu et al., 2019; Wang J. et al., 2019; Pham et al., 2020). Similarly, the positive effect of supplementation of the OA on performance was comparable to AGP in the present study. And grower phase may be the key period for the OA where they exert the growth-promoting effects. Supplementation of OA in the diet was reported that could reduce pH in the gastrointestinal tract, increase the activity of digestive enzymes and improve apparent ileal digestibility of broilers (Hernández et al., 2006; Garcia et al., 2007; Ao et al., 2009). Additionally, our results also indicated that feeding OA improved intestinal morphology presenting a higher VH of ileum, which was consistent with previous studies (Liu et al., 2017; Yang et al., 2018). Superior intestinal morphology suggested an improved intestinal nutrient digestibility and absorption capacity and might further contribute to subsequent improvement in the growth performance (Montagne et al., 2003). Conversely, a few of studies suggested that the OA failed to improve growth performance and intestinal morphology (Fascina et al., 2012). These inconsistencies may be related to differences in the composition and inclusion levels used, diet type, environmental conditions, and breed (Khan and Iqbal, 2016). Therefore, it’s necessary to use more detailed parameters to evaluate AGP alternatives.

The intestinal barrier regulated by tight junction proteins performs the crucial role of defense against the passage of pathogens and antigens into the intestinal epithelium (Broom, 2018). Intestinal tight junction barrier disruption leads to endogenous infection and inflammation and tissue damage as well as decrease of nutrient absorption (Peterson and Artis, 2014). In the present study, both OA and AGP up-regulated mRNA expressions of intestinal barrier genes relative to the NC group, such as Claudin-1, Claudin-3, and ZO-1, which were consistent with previous studies (Stefanello et al., 2019; Pham et al., 2020). As the most important components of the intestinal epithelial barrier, Claudin-1, and Claudin-3 play important roles in barrier formation and paracellular selectivity (Suzuki, 2013). The high expression of Claudin-1 resulted in increased tightness and decreased permeability of epithelial cells in chickens (Awad et al., 2017). ZO-1 is among important molecules that interact directly with claudins and provide a scaffold that facilitates regulation of the expression (Bauer et al., 2010). Therefore, changes in the expression of Claudins-1 and Claudins-3 are accompanied by up-regulated the expression of ZO-1 in OA and PC groups. In contrast, Claudin-2 is a pore-forming tight junction protein (Suzuki, 2013). The up-regulation of Claudin-2 expression was reported in inflammatory intestinal disease, and it was usually considered an indicator of inflammation (Turner, 2009). Therefore, the higher mRNA expression of Claudin-2 suggested that AGP might have a potential negative impact on intestinal immune homeostasis. Meanwhile, the higher and lower GC of jejunum were observed in OA and PC groups, respectively, suggesting that broilers fed with OA had more homeostatic and health intestinal environment than that with AGP. Because the Mucin-2 covered intestinal epithelial surface can be secreted by goblet cells and exerts a major role in protecting the intestinal epithelium from infection and maintaining intestinal mucosal barrier integrity, immune homeostasis and gut health (Kim and Ho, 2010). Taken together, OA could improve the integrity of the intestinal epithelium, consequently generating a more friendly gut environment than AGP, which could help defend against pathogen infection.

The alterations in intestinal microbiota and metabolites such as SCFAs can both positively and negatively affect the intestinal barrier function (Yu et al., 2019; Liu et al., 2020). Therefore, we speculate that the shift of microbiota and high concentrations of butyrate in the present study may be the factors accounting for the regulation of intestinal barrier function. In attempts to better understand the mechanism and validate our hypothesis, we analyzed the cecal microbiota which contains the vast majority of intestinal bacteria and stronger fermentation of chicken intestinal microbiota (Pourabedin and Zhao, 2015). Data from analysis of β-diversity corroborate our initial hypothesis that OA could change microbial structure (ANOSIM: *R* = 0.197, *P* = 0.010). Similar to the previous reports (Hu et al., 2019; Pham et al., 2020), there was no difference in the Shannon and Simpson indices used to assess the microbiota diversity on day 42. However, a decreasing trend in the ACE and Chao1 estimators reflecting the microbiota richness was observed in the OA group (0.05 < *P* < 0.10). Generally, there is the competition between host animal and microbiota for diets nutrients due to the abundant colonization of bacteria in the gastrointestinal tract (Suiryanrayna and Ramana, 2015). Therefore, it is crucial to regulate the population balance of the intestinal bacteria besides to control potential pathogenic bacteria.

Similar to previous observation in chickens, the phyla Firmicutes, Bacteroidetes, and Proteobacteria were the dominant bacteria in control broilers (Oakley et al., 2014). In the current study, higher abundance of Firmicutes at expense of Bacteroidetes demonstrated that OA shift cecal microbiome composition of birds. Besides reducing buffering capacity of the feed to prevent the colonization of pathogen (Suiryanrayna and Ramana, 2015), OA also have bacteriostatic and bactericidal capacity. Due to differences in cell wall structure, the non-dissociated OA (formic, acetic, propionic, and sorbic acids) can exclusively perforate the cell wall of gram negative bacteria and release hydrogen ions, which consequently reduce the intracellular pH and disrupt directly the normal physiology of cytoplasm including replication and protein synthesis on the cellular level (Ricke, 2003; Khan and Iqbal, 2016; Dittoe et al., 2018). Therefore, the abundance of *Coprobacter* and *Bacteroides* belonging to gram negative bacteria and dominant genus of Bacteroidetes were decreased, which lead to higher Firmicutes to Bacteroidetes ratio in the OA group. Furthermore, animals intestinal microbiota with the higher Firmicutes to Bacteroidetes ratio had improved nutrient digestibility and greater body weight in previous studies (Ley et al., 2006; Singh et al., 2013). Therefore, higher abundance of Firmicutes at expense of Bacteroidetes might contribute to the nutrient utilization, consequently improving the growth performance of broilers fed with OA in the present study.

Further analysis revealed that several species were identified as biomarkers to distinguish microbiota of broilers among groups. Consistent with previous studies (Lopetuso et al., 2013; Palamidi and Mountzouris, 2018; Hu et al., 2019), we found that Ruminococcaceae, Christensenellaceae, and Peptococcaceae producing SCFAs were enriched in the OA group. Thus, one potential explanation for increased digesta SCFAs may be due to increase the abundance or numbers of SCFAs-producing bacteria in the cecum of broilers fed with OA. Ruminococcaceae members considered beneficial autochthonous taxa has been well illustrated to be responsible for the degradation of diverse polysaccharides and fibers (Hooda et al., 2012), and then producing SCFAs to provide energy to the intestinal epithelium. Therefore, numerous studies indicated that Ruminococcaceae was positively correlated with gene functions related to energy metabolism, intestinal morphology of chickens (Zhang et al., 2018; Hu et al., 2019; Dai et al., 2020), which are indispensable for the hosts. Similarly, Ruminococcaceae was showed highly positive correlations with the BW and the mRNA level of ZO-1 in the present study. Recent studies reported that Christensenellaceae was associated with the health of human (Mancabelli et al., 2017; Waters and Ley, 2019; Pittayanon et al., 2020). And genus *Christensenellaceae_R-7_group* was related to the production of Mucin-2 (Xie et al., 2020), which was consistent with increased the GC of jejunum in the OA group. Besides, Peptococcaceae was overrepresented in the microbiota of the probiotics group in the previous study (Li et al., 2019), and members of this family have been shown to be key SCFAs producers (Ye et al., 2017). A positive correlation of *norank_f_Peptococcaceae* with tight junction protein mRNA level also was found (Geng et al., 2019). To sum up, the changes of microbiota by the OA supplementation exerted an important role in improving growth performance and intestinal barrier of broilers in the present study.

Additionally, we found that AGP and OA had a distinct impact on cecal microbiota. Previous studies showed that dietary supplementation with AGP altered the composition of chicken microbiota, mainly reducing the abundance of Firmicutes and *Lactobacillus* in the gastrointestinal tract (Lin et al., 2013), and increasing the abundance of Lachnospiraceae (Broom, 2018), which were consistent with our study. As the SCFAs producers, a positive correlation has been found between the abundance of Lachnospiraceae and growth performance in broilers (Stanley et al., 2016; Hu et al., 2019). However, the Lachnospiraceae was also associated with metabolic disorders including glucose and lipid metabolism in the obese mice and might impact human health (Kameyama and Itoh, 2014; Rodrigues et al., 2017). The increased *Eisenbergiella* genera of Lachnospiraceae was also found in the gut of mice infected with *Echinococcus*, suggesting it may be associated with a Th2 response (Bao et al., 2018). However, there is very limited information on biological function of this genus. In addition, *Coprobacter* was also enriched in the PC group, which was consistent with previous studies (Danzeisen et al., 2011). Interestingly, recent studies reported that chickens with high abundance of *Coprobacter* in cecum had more antibiotic resistance genes using metagenome sequencing (Kumar et al., 2020), suggesting that the high abundance of *Coprobacter* might lead to the antibiotic resistance. Otherwise, dietary AGP addition also triggered increase in the abundances of *Bacteroides* and was accompanied by decreased Firmicutes to Bacteroidetes ratio compared with the OA group. Due to inhibition of transglycosylase activities and cell wall synthesis, flavomycin primarily inhibits gram positive bacteria (Pfaller, 2006). Therefore, the significantly reduced abundances of Ruminococcaceae and Christensenellaceae were observed in the PC group, which lead to lower Firmicutes to Bacteroidetes ratio. In fact, the Firmicutes to Bacteroidetes ratio is considered as a biomarker of superior gastrointestinal function, and can be indicative of eubiosis conditions in the intestine (Cheng et al., 2017). In addition, the previous study of our team found that *Bacteroides* was positively related with the expression of pro-inflammatory cytokines IL-1β, IL-8, and TNF-α in the ileum of laying hens (Wang W. W. et al., 2019). Thus, considering similar effects on the growth performance, OA could provide a more homeostatic and healthy intestinal microecology than AGP for animals, which suggested OA would be a suitable alternative to AGP.

## Conclusion

Supplemental OA improved growth performance, intestinal morphology and barrier function, which might be attributed to the changes of intestinal microbiota, particularly the enrichment of SCFAs-producing bacteria. Simultaneously, OA as a suitable alternative to AGP could provide a more homeostatic and healthy intestinal microecology than AGP for animals, whereas AGP caused the dysbiosis of intestinal barrier and microbiota. The conclusions can expand our fundamental knowledge concerning the regulatory role of OA on the intestinal health and growth performance in the poultry production as the AGP alternatives.

## Data Availability Statement

The raw data supporting the conclusions of this article will be made available by the authors, without undue reservation.

## Ethics Statement

The animal study was reviewed and approved by the Animal Care and Use Committee of the Feed Research Institute of the Chinese Academy of Agricultural Sciences.

## Author Contributions

JW, Y-mH, and Y-yW conceived and designed the experiments. DD performed the animal experiments, analyzed the data, and wrote the manuscript. JW, G-hQ, KQ, H-jZ, and S-gW supervised and provided continuous guidance for the experiments. All authors have discussed the results and reviewed the manuscript.

## Conflict of Interest

The authors declare that the research was conducted in the absence of any commercial or financial relationships that could be construed as a potential conflict of interest.
